# Diffuse Idiopathic Pulmonary Neuroendocrine Cell Hyperplasia Mimicking Lung Cancer: A Case Report

**DOI:** 10.7759/cureus.98920

**Published:** 2025-12-10

**Authors:** Dimitrios Charalampous, Sofoklis Mitsos, Vasileios Leivaditis, Efstratios Koletsis, Periklis Tomos

**Affiliations:** 1 Department of Thoracic Surgery, National and Kapodistrian University of Athens, Attikon University Hospital, Athens, GRC; 2 Department of Cardiothoracic and Vascular Surgery, Westpfalz-Klinikum, Kaiserslautern, DEU; 3 Department of Cardiothoracic Surgery, General University Hospital of Patras, Patras, GRC

**Keywords:** diffuse idiopathic pulmonary neuroendocrine cell hyperplasia (dipnech), lobectomy, lung tumor, neuroendocrine cells, pulmonary carcinoid tumor, solitary pulmonary nodule, tumorlets

## Abstract

Diffuse idiopathic pulmonary neuroendocrine cell hyperplasia (DIPNECH) is an extremely uncommon and incompletely elucidated pulmonary condition arising from neuroendocrine cells within the airway epithelium. We report the case of a 65-year-old woman who presented to our thoracic surgery department with a history of hemoptysis following the incidental discovery of a solitary pulmonary nodule in the right middle lobe, highly suspicious for lung cancer. After a thorough diagnostic workup, including positron emission tomography-computed tomography (PET-CT), our multidisciplinary team recommended a right middle lobectomy. The histopathological examination of the resected specimen confirmed DIPNECH without evidence of an associated carcinoid tumor. Given the potential for progression to neuroendocrine neoplasm, the development of evidence-based management protocols for DIPNECH remains essential. This case highlights the atypical and often silent presentation of this rare lung pathology and emphasizes the importance of considering DIPNECH in the differential diagnosis of asymptomatic patients presenting with solitary pulmonary nodules.

## Introduction

Diffuse idiopathic pulmonary neuroendocrine cell hyperplasia (DIPNECH) is an extremely rare precancerous lung disease originating from pulmonary neuroendocrine cells (PNECs), which are also known as Kulchitsky or Feyrter cells. PNECs are sparsely distributed among other lung epithelial cells across the entire length of the mammalian respiratory tract and, in some regions, are organized into clusters termed neuroepithelial bodies. Although their role is not yet completely understood, PNECs are considered to be much more prevalent in the lungs during fetal development and in utero life, while their number is constantly declining over the course of aging, consisting of less than 1% of all lung cells during adulthood [1]. In light of this fact, it is believed that PNECs play a crucial role in the paracrine regulation during lung development, acting as O_2_, CO_2_, and mechanical stress sensors that release amine hormones (serotonin, dopamine, and norepinephrine), peptide hormones (bombesin, calcitonin, and leu-enkephalin), vasoactive intestinal peptide (VIP) and substance P, affecting the contractility of smooth muscle cells and maintaining appropriate pulmonary circulation and oxygen perfusion [2,3].

DIPNECH constitutes a rare medical disorder, first recognized in the early 1950s, but it was not until 1992 that Aguayo et al. published a case series of six patients suffering from this pulmonary pathology, linking DIPNECH to hyperplasia of PNECs. In this landmark scientific paper, the authors described six nonsmoking patients who presented with dyspnea, cough, abnormal pulmonary function tests (PFTs), and multiple pulmonary nodules composed of proliferated PNECs, while peribronchial fibrosis was also identified [4]. According to the fifth edition of the World Health Organization (WHO) Classification of Thoracic Tumors, published in 2021, DIPNECH is also acknowledged as a precursor lesion for carcinoid tumors, mostly typical ones, while there is no connection with high-grade pulmonary neuroendocrine neoplasms, such as small cell lung cancer [5]. However, DIPNECH does not precede in all cases of carcinoid tumors; therefore, there is weak evidence even about the connection between DIPNECH and this type of tumor [6].

To date, there are fewer than 300 published cases of DIPNECH worldwide in the form of case reports or small case series, highlighting that this clinical entity is poorly understood [7]. Herein, with this case report, we aim to underscore the unusual and asymptomatic presentation of this pulmonary disease that can potentially give rise to a neuroendocrine neoplasm if a prompt therapeutic approach is not established.

## Case presentation

**Figure 1:**
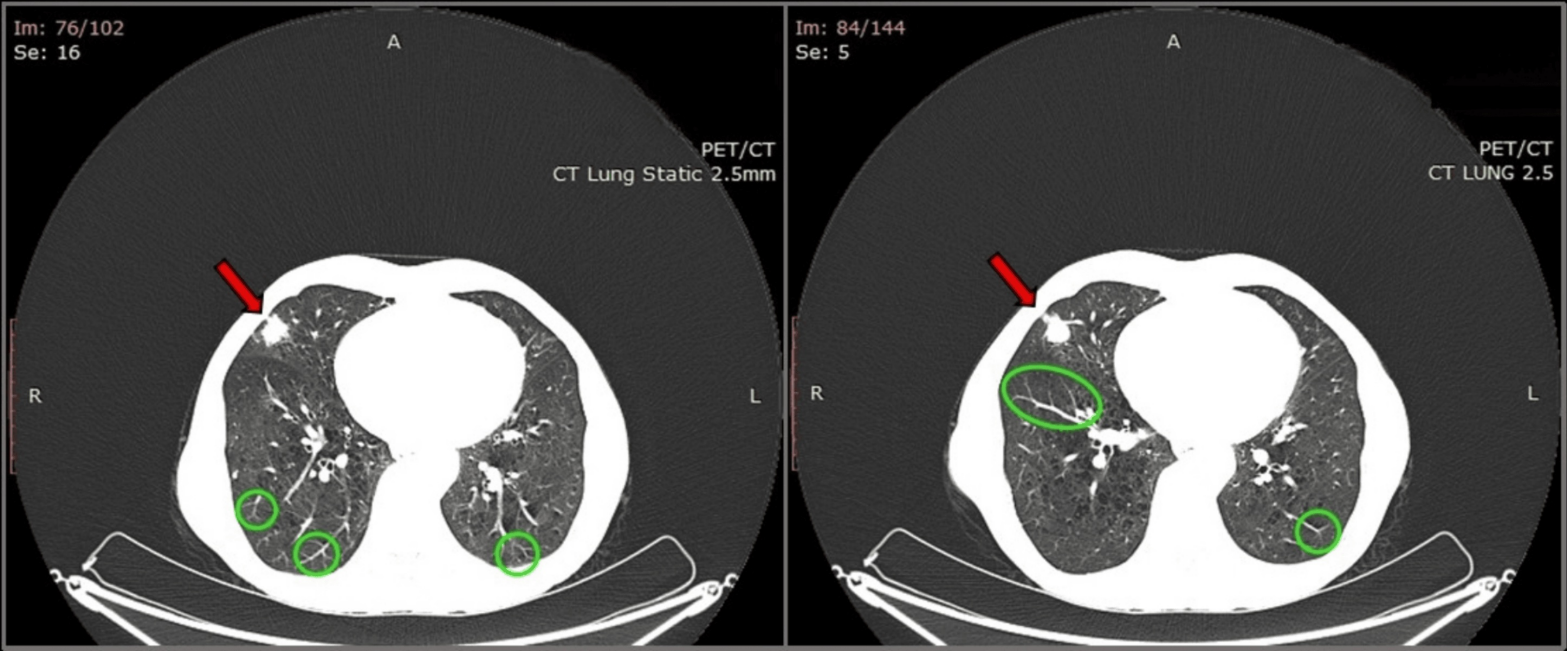
CT Scan of the Lungs Revealing a Middle Lobe Nodule With Multiple Micronodules Chest CT scan lung window images reveal a noncalcified nodular lesion with irregular margins, measuring 18 mm in diameter, located in the lateral segment of the middle lobe, highly suspicious for lung cancer (red arrows). Furthermore, multiple bilateral micronodules, along with tree-in-bud opacities, are observed (green circles) PET/CT: positron emission tomography/computed tomography

A 65-year-old woman presented to our thoracic surgery department for further evaluation of a pulmonary nodule located in the right middle lobe, incidentally found in a chest computed tomography (CT) scan. The woman had previously been admitted to another hospital because of hemoptysis. In order to rule out pulmonary embolism, she underwent a CT pulmonary angiogram (CTPA) scan, which highlighted the existence of a nodular lesion with irregular margins, 1.8 cm in diameter, in the lateral segment of the middle lobe. The abovementioned nodule was characterized as contrast-enhanced, highly suspicious for lung cancer. In addition, during the bronchoscopy performed on the following days, a small endobronchial blood clot was found in the posterior segment of the right upper lobe and the basal segments of the right lower lobe without evidence of ongoing hemorrhage. The patient was then recommended to undergo a positron emission tomography (PET)-CT scan for further characterization of her pulmonary nodule, while she was referred to our clinic with the aim of possible surgical management (Figure 1).

The woman presented to our clinic with her medical record from the referring hospital, and she denied cough, dyspnea, wheezing, or chest discomfort in the last few months. According to her account, she had neither lost weight nor experienced fever or night sweats. Her medical background was insignificant, and there was no tuberculosis history. The woman reported a previous 60-pack-year smoking history, but she denied smoking for a month before presentation. Additionally, there was no evidence of environmental or occupational exposure to hazardous chemicals. She denied taking any medication and had no allergies and personal or family history of pulmonary disease. On physical examination, normal respiratory sounds were auscultated, while the examination of other systems was unremarkable. Routine admission blood test and initial arterial blood gas analysis were also within normal limits.

**Figure 2:**
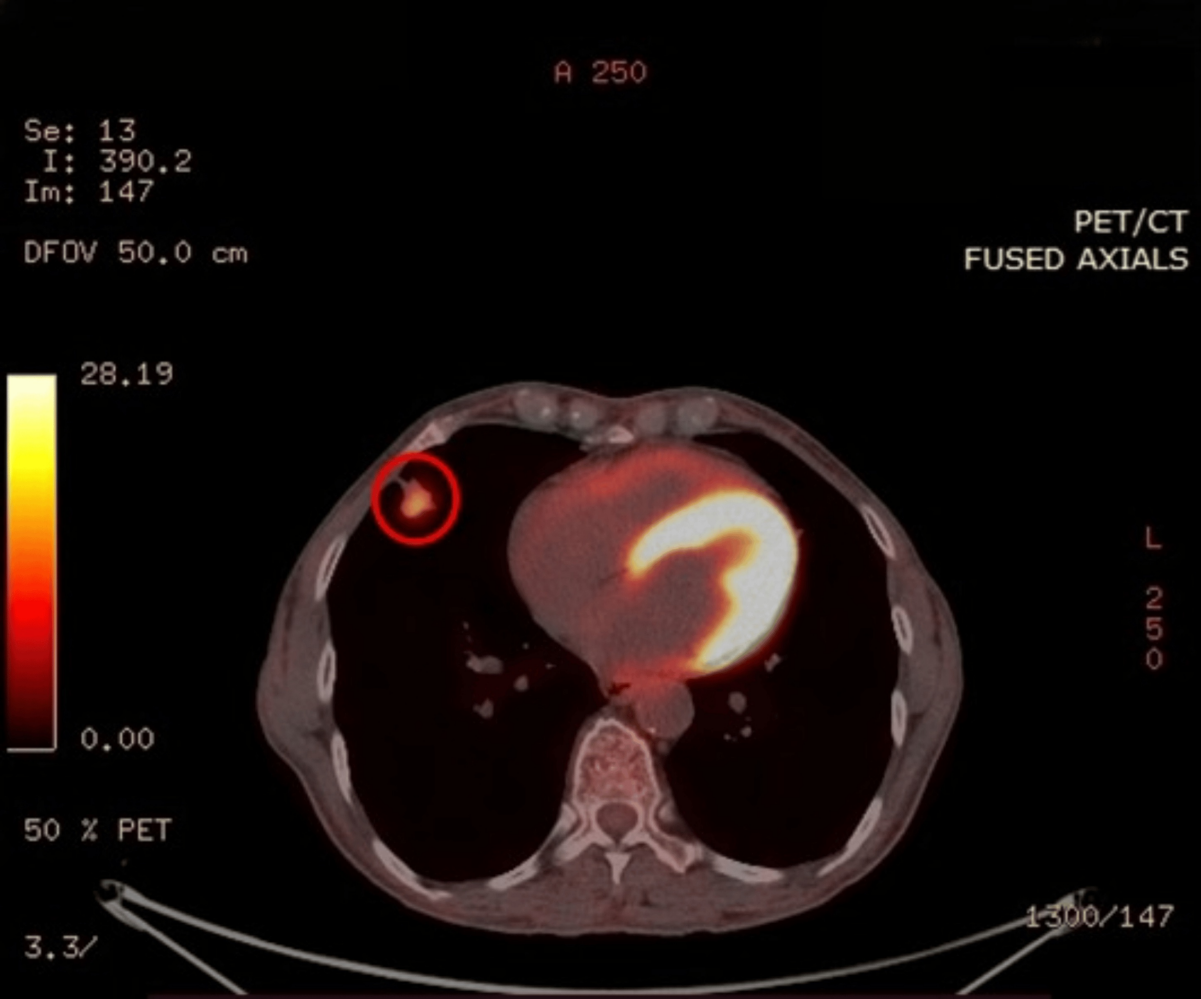
Metabolically Active Pulmonary Nodule on 18F-FDG PET-CT Scan 18F-FDG PET-CT image demonstrates the metabolically active 18 mm pulmonary nodule with a SUVmax of approximately 6, located in the lateral segment of the right middle lobe (red circle) 18F-FDG, 18F-fluorodeoxyglucose; PET-CT, positron emission tomography-computed tomography; SUVmax, maximum standardized uptake value

On admission, she submitted an 18F-fluorodeoxyglucose (18F-FDG) PET-CT scan, which was positive for a nodular, metabolically active lesion, 18x15 mm in dimension, with a standardized 18F-FDG uptake value (maximum standardized uptake value {SUVmax}) of 6.2 (Figure 2). The nodule was thought to be in close contact with the pleura and the horizontal fissure. No enlarged or metabolically active lymph nodes were observed in the hilar region or mediastinum. It is also worth mentioning that multiple bilateral nodular and tree-in-bud opacities were identified, along with possible subtle air trapping, findings suggestive of small airway disease. Considering that the results of the PET-CT scan were highly suspicious for lung cancer, this case was discussed thoroughly during the weekly multidisciplinary oncology council, and right middle lobectomy was considered the appropriate management plan for this patient. In view of surgery, the patient underwent PFTs with a forced expiratory volume in one second (FEV_1_) of 1.98L, 89% of the predicted value, and a diffusing capacity for carbon monoxide (DL_CO_) of 75%. In addition, a head CT scan was performed with no evidence of metastasis.

A middle lobectomy with the dissection of adjacent pulmonary lymph nodes was performed, and the specimen was sent to the histopathology unit. Histopathological evaluation revealed a lesion with indistinct margins and a white-gray color, measuring approximately 13 mm in diameter, as seen on macroscopic examination. Microscopically, the lesion demonstrated chronic inflammatory infiltrates, along with nodular and linear proliferative hyperplastic neuroendocrine lung cells. Immunohistochemical staining was positive for cytokeratin AE1/AE3, chromogranin, synaptophysin, cluster of differentiation 56 (CD56), and thyroid transcription factor 1 (TTF-1), ascertaining the neuroendocrine origin of this nodule. The cellular proliferation index Ki-67 was less than 5%. Additionally, adjacent neuroendocrine cell aggregations less than 5 mm in diameter were observed, highlighting the existence of tumorlets. No carcinoid tumor in the specimen was identified. All the dissected lymph nodes were reactively hyperplastic with no evidence of metastasis. Having an uneventful recovery, the patient was discharged in good overall health, and she was advised to close and lifelong monitoring by a pulmonologist and an oncologist.

## Discussion

According to the WHO, DIPNECH is defined as a disseminated proliferation of scattered single cells and small nodules (neuroendocrine bodies) or linear proliferations of PNECs confined to the bronchial and bronchiolar epithelium. By definition, in DIPNECH, neuroendocrine cells do not penetrate the basement membrane and form nodular aggregates smaller than 5 mm. If a disruption of basement membrane integrity is observed, tumorlets (<5 mm) or carcinoid tumors (>5 mm) should be suspected [6,8]. Due to the rarity of DIPNECH cases and the scarce scientific evidence available, its true incidence and prevalence remain unknown. In general, hyperplasia of PNECs can be seen in three different settings. Firstly, the reactive proliferation of pulmonary neuroendocrine cell hyperplasia (NECH) may be encountered as a result of hypoxia stimuli, such as chronic pulmonary diseases, smoking-related interstitial lung disease, pulmonary fibrosis, bronchopulmonary dysplasia, cystic fibrosis, bronchiectasis, chronic high-altitude exposure, tobacco smoke exposure, and chronic obstructive pulmonary disease (COPD) [9]. Secondly, hyperplasia of PNECs has been reported in up to 75% of lung specimens examined histopathologically after resection for carcinoid tumors, in the area adjacent to the primary lesion. Thirdly, when no predisposing factors, such as those described above, are present, DIPNECH should be suspected, especially in the clinical context of obstructive airway disease [10,11].

After the outstanding work of Aguayo et al. in 1992, the WHO classified DIPNECH as a preinvasive lesion for the first time in 1999 [4]. This pulmonary disease mostly occurs in women during the sixth and seventh decades of life. It has been demonstrated that there is a strong female predisposition, resulting from the fact that the female-to-male ratio is 9-10:1. While no such connection is observed for reactive NECH, it has not yet been explained why DIPNECH is markedly more prevalent in women. Consequently, a potential genetic or hormonal role in this disease process has not yet been elucidated [12]. Additionally, most patients are nonsmokers, evidence that conflicts with the known proliferative effect of smoke on PNECs. Approximately 50% of patients are asymptomatic at the time of diagnosis, while the most common symptoms are mainly cough, dyspnea that may worsen over time due to obstructive airway disease, and wheezing [12]. Home oxygen requirement or hemoptysis may also be present. The mean interval between the onset of symptoms and the diagnosis of DIPNECH disease is estimated at 10 years, illustrating that this pulmonary entity still remains a diagnostic and therapeutic challenge for clinicians [13].

Based on the 2021 WHO Classification of Thoracic Tumors, DIPNECH is further classified into two distinct entities: clinical and pathological [5]. Clinical DIPNECH refers to symptomatic patients with cough or breathlessness due to mild or severe airway obstruction and CT findings suggestive of DIPNECH. On the other hand, pathological DIPNECH refers to patients with no symptoms, in whom the diagnosis is established after lung biopsy for other reasons. At this point, it is pertinent to note that a definite DIPNECH diagnosis requires histopathological assessment. For this reason, surgical lung biopsy is widely recognized as the “gold standard” [12]. According to Marchevsky et al., histopathological criteria for diagnosis require the presence of ≥5 PNECs within the basement membrane of ≥3 bronchioles, as well as the identification of ≥3 carcinoid tumorlets [14]. However, these criteria have not yet been endorsed by the WHO. To date, no pathognomonic CT findings for DIPNECH have been established, but characteristic imaging features include multiple pulmonary nodules and mosaic attenuation with air trapping, especially pronounced on expiratory chest CT when airway obstruction is evident [15]. In our case, expiratory chest CT was not performed, and consequently, air trapping could not be definitively detected [5].

Nowadays, management strategies for DIPNECH include pharmacologic interventions or surgery. Given the lack of standardized and widely accepted guidelines, the therapeutic plan is individualized, taking into consideration a variety of factors, such as patient status, comorbidities, life expectancy, and symptoms. For symptomatic patients, inhaled or oral corticosteroids, along with bronchodilation, may result in improvement, while octreotide (a somatostatin analogue) or everolimus (an inhibitor of mammalian target of rapamycin {mTOR}) has been used in cases where somatostatin receptors are expressed or the mTOR pathway is activated. In contrast, for patients with no symptoms, a “watch and wait” strategy is preferred with annual or biennial surveillance [16]. Last but not least, in cases where the disease is localized or a DIPNECH diagnosis cannot be established, such as in our patient, surgical treatment with lobectomy or wedge resection is considered the gold standard [17].

In our case, the patient was completely asymptomatic according to her account. In addition, while radiological findings of DIPNECH typically include multiple nodules and tree-in-bud opacities, the presence of a large pulmonary nodule, especially when it is metabolically active on PET-CT scan, poses a significant diagnostic challenge. In this context, differential diagnosis necessarily includes neoplastic, metastatic, infectious, and autoimmune processes. Our case emphasizes the importance of considering DIPNECH in the differential diagnosis of asymptomatic patients presenting with solitary pulmonary nodules. To date, 2.5 years after diagnosis, the patient continues to demonstrate clinical improvement while adhering to inhaler therapy. Surgical management remains the appropriate therapeutic approach, as the possibility of lung cancer could not otherwise be excluded. Notably, in most of the DIPNECH cases, bilateral nodules, as demonstrated in high-resolution chest CT scan, are small in diameter (4-10 mm), whereas larger lesions suggest the presence of carcinoid tumors, making our patient’s presentation particularly unusual [10,18].

## Conclusions

In summary, diffuse idiopathic pulmonary neuroendocrine cell hyperplasia is an extremely rare yet frequently misdiagnosed medical entity. Given its rarity, there are no established diagnostic or therapeutic guidelines, posing a challenging task for clinicians to handle patients with this condition. Thanks to advancements in medical imaging techniques, DIPNECH is expected to be increasingly detected in the following years, emphasizing that more research is definitely required for this pulmonary disease in order to implement evidence-based medicine. Moreover, in light of the potential progression to carcinoid tumor, the development of a research-based therapeutic approach for DIPNECH is of utmost importance. We hope that this particular case contributes to a more comprehensive understanding of DIPNECH presentation and diagnosis, highlighting the necessity of formulating standardized management protocols.
